# Elevated amyloid beta disrupts the nanoscale organization and function of synaptic vesicle pools in hippocampal neurons

**DOI:** 10.1093/cercor/bhac134

**Published:** 2022-04-03

**Authors:** Luca Biasetti, Stephanie Rey, Milena Fowler, Arjuna Ratnayaka, Kate Fennell, Catherine Smith, Karen Marshall, Catherine Hall, Mariana Vargas-Caballero, Louise Serpell, Kevin Staras

**Affiliations:** Sussex Neuroscience, School of Life Sciences, University of Sussex, Brighton, BN1 9QG, United Kingdom; Sussex Neuroscience, School of Life Sciences, University of Sussex, Brighton, BN1 9QG, United Kingdom; National Physical Laboratory, Middlesex, TW11 0LW, United Kingdom; Sussex Neuroscience, School of Life Sciences, University of Sussex, Brighton, BN1 9QG, United Kingdom; Sussex Neuroscience, School of Life Sciences, University of Sussex, Brighton, BN1 9QG, United Kingdom; Faculty of Medicine, University of Southampton, SO17 1BJ, United Kingdom; Sussex Neuroscience, School of Life Sciences, University of Sussex, Brighton, BN1 9QG, United Kingdom; Sussex Neuroscience, School of Life Sciences, University of Sussex, Brighton, BN1 9QG, United Kingdom; Sussex Neuroscience, School of Life Sciences, University of Sussex, Brighton, BN1 9QG, United Kingdom; Sussex Neuroscience, School of Psychology, University of Sussex, Brighton, BN1 9QH, United Kingdom; School of Biological Sciences, University of Southampton, Highfield Campus, Southampton SO17 1BJ, United Kingdom; Sussex Neuroscience, School of Life Sciences, University of Sussex, Brighton, BN1 9QG, United Kingdom; Sussex Neuroscience, School of Life Sciences, University of Sussex, Brighton, BN1 9QG, United Kingdom

**Keywords:** amyloid beta, transmission, vesicle, hippocampus, synapse

## Abstract

Alzheimer’s disease is linked to increased levels of amyloid beta (Aβ) in the brain, but the mechanisms underlying neuronal dysfunction and neurodegeneration remain enigmatic. Here, we investigate whether organizational characteristics of functional presynaptic vesicle pools, key determinants of information transmission in the central nervous system, are targets for elevated Aβ. Using an optical readout method in cultured hippocampal neurons, we show that acute Aβ42 treatment significantly enlarges the fraction of functional vesicles at individual terminals. We observe the same effect in a chronically elevated Aβ transgenic model (APP_Sw,Ind_) using an ultrastructure-function approach that provides detailed information on nanoscale vesicle pool positioning. Strikingly, elevated Aβ is correlated with excessive accumulation of recycled vesicles near putative endocytic sites, which is consistent with deficits in vesicle retrieval pathways. Using the glutamate reporter, iGluSnFR, we show that there are parallel functional consequences, where ongoing information signaling capacity is constrained. Treatment with levetiracetam, an antiepileptic that dampens synaptic hyperactivity, partially rescues these transmission defects. Our findings implicate organizational and dynamic features of functional vesicle pools as targets in Aβ-driven synaptic impairment, suggesting that interventions to relieve the overloading of vesicle retrieval pathways might have promising therapeutic value.

## Introduction

Alzheimer’s Disease (AD) is the most common form of dementia, and the disease progression is directly correlated with neuronal and synaptic loss (DeKosky and Scheff 1990; Chen et al. 2019). AD is characterized by the accumulation of intracellular tau in neurofibrillary tangles and extracellular deposition of amyloid beta (Aβ) in amyloid plaques. Aβ peptides of lengths 38–46 are produced via cleavage of amyloid precursor protein (APP) by β- and γ-secretases, and increased levels of Aβ42, in particular, are implicated in AD (Benilova et al. 2012). The presence of amyloid plaques is an established diagnostic criterion for AD, but it is generally agreed that the existence of small, soluble oligomeric forms of Aβ are more closely aligned with disease severity (Lue et al. 1999; McLean et al. 1999; Wang et al. 1999). The impact of oligomeric Aβ on postsynaptic mechanisms (Lacor et al. 2004; Molnár et al. 2004; Koffie et al. 2009), including the impairment of forms of long-term plasticity (Li et al. 2009; Tu et al. 2014), has been reported in numerous studies, but accumulating evidence suggests that presynaptic substrates are particularly important in understanding the nature of synaptic dysfunction in AD (Kokubo et al. 2005; Chakroborty et al. 2012; Sokolow et al. 2012; Park et al. 2013; Lazarevic et al. 2017; Marsh and Alifragis 2018; Fagiani et al. 2019). Consistent with this, APP is presynaptically ubiquitous (Groemer et al. 2011; Laßek et al. 2016; Weingarten et al. 2017) and findings indicate that Aβ oligomers are readily internalized and accumulate at presynaptic terminals (Russell et al. 2012) more than at postsynaptic structures (Yu et al. 2018). Moreover, Aβ42 oligomers have been shown to interact with key presynaptic proteins, including syntaxin 1a (Yang et al. 2015), synaptophysin (Russell et al. 2012), synapsin (Marsh et al. 2017; Park et al. 2017), and dynamin (Kelly and Ferreira 2007).

A presynaptic locus for Aβ42 action has particular relevance because these highly specialized compartments contain the neurotransmitter-containing synaptic vesicles (SVs) responsible for regulated point-to-point information signaling. SVs are morphologically identical, but the total vesicle population at a single terminal is subdivisible into functionally different populations, or pools, whose properties, including their size, physical organization, availability for release, and kinetics of recycling, are critical determinants of synaptic strength (Betz and Bewick 1992; Ryan and Smith 1995; Murthy et al. 1997; Harata, Pyle, et al. 2001; Schikorski and Stevens 2001; Rizzoli and Betz 2004; Branco et al. 2008; Branco and Staras 2009; Marra et al. 2012; Park et al. 2012; Rey et al. 2015, 2020). One such vesicle population, the total recycling pool (TRP) (Denker and Rizzoli 2010; Alabi and Tsien 2012; Fowler and Staras 2015), represents the full complement of vesicles that engage in activity-evoked turnover. Thanks to the role of these vesicles in contributing to information transfer and their known modulation in forms of plasticity (Murthy et al. 2001; Zakharenko et al. 2001; Thiagarajan et al. 2005; Bayazitov et al. 2007; Kim and Ryan 2010; Ratnayaka et al. 2012; Rey et al. 2020), their potential relevance as targets for disease-related dysfunction is significant (Li and Kavalali 2017; Melland et al. 2021). Work to date offers a complex picture of the action of Aβ on vesicular function, likely reflecting differences in AD models and treatment conditions. In general, brief, low concentrations of Aβ have often been reported to act as a positive regulator of vesicle release (Puzzo et al. 2008; Abramov et al. 2009; Russell et al. 2012; Yang et al. 2015; Lazarevic et al. 2017; Palmeri et al. 2017; Marsh and Alifragis 2018; Anni et al. 2021; Taylor et al. 2021), while higher level exposure can have excitotoxic effects, negatively influencing vesicle recycling and potentially contributing to a role in limiting signaling capacity (Kelly and Ferreira 2007; Park et al. 2013, 2017; Lazarevic et al. 2017; Marsh et al. 2017).

Here, we investigated how organizational principles of SV pools might be impacted in elevated Aβ conditions and whether these could explain observed functional defects. Using a synapse-specific optical readout in boutons of dissociated hippocampal neurons, we show that 4–5-day incubation with Aβ42 oligomers significantly increases the ratio of recycling to nonrecycling vesicles. In a transgenic mouse model of chronically elevated Aβ42 (APP_Sw,Ind_) (Jankowsky et al. 2005), a function-ultrastructure approach demonstrates that the TRP fraction is similarly enlarged and that an organizational signature of aberrant recycling is seen in which retrieved SVs accumulate at the peri-active zone (AZ), the putative sites of endocytosis. Using the glutamate reporter, iGluSnFR, we show that signaling deficits emerge in Aβ42-treated synapses in hippocampal cultures during repeated stimulation, which is consistent with a use-dependent impairment of vesicle turnover. Levetiracetam (LEV), an antiepileptic agent that serves to dampen synaptic hyperactivity, partially rescues these activity-history dependent transmission deficits and restores ongoing signaling capacity. Our findings suggest that elevated levels of Aβ42 have effects on the availability, recycling, and trafficking of functional SV pools that could underlie key aspects of AD-related synaptotoxic effects.

## Materials and methods

### Experimental model and subject details

Experiments were carried out in accordance with the UK-Animals (Scientific Procedures) Act 1986 and satisfied the local institutional regulations at the University of Sussex and the University of Southampton. The project was given ethical approval by the local Ethical Review Committee (University of Sussex: ARG/1/4). Rat primary hippocampal cultures were prepared from P_0_-P_1_ pups sacrificed using Schedule 1 methods. The AD model used was a double transgenic mouse/human APP695 containing Swedish (KM570/571NL) and Indiana (V617F) mutations, with a tetracycline transactivator for controllable doxycycline-responsive APP transgene expression (Jankowsky et al. 2005) (https://www.jax.org/strain/007051). Mice expressed the APP_Sw,Ind_ transgene from birth and were used at 3 months; previous studies have demonstrated that such animals display higher levels of Aβ protein compared to matched controls (Sri et al. 2019). Animals originated from Joanna Jankowsky (Baylor College of Medicine, United States) via Dr Mariana Vargas-Caballero (University of Southampton) under a material transfer agreement between Joanna Jankowsky and Louise Serpell. Home Office project licenses were PPL 70-8762 (Catherine Hall, Sussex) and PPL 30/3091 (Mariana Vargas-Caballero, Southampton).

### Aβ preparation

Aβ(1–42) was prepared using established methods shown to generate reproducible, neurotoxic oligomeric species with predominantly random coil structure and uniform morphology (Marshall et al. 2016, 2020). Lyophilized Aβ_42_ (CAT# A-1163-02, 0.2 mg from rPeptide, United States, stored at −20 °C) was resuspended by adding 200 μL of HFIP (1 mg/mL) to the vial, which was then vortexed for 1 min before undergoing sonication for 5 min (50/60 Hz). Resuspended Aβ was then evaporated by passing low flow nitrogen gas until a transparent HFIP film was left at the bottom of the vial. Two hundred microliters of DMSO were subsequently added to the vial before vortexing (1 min) and sonicating (1 min). Zeba 7K MWCO (ThermoScientific) 2-mL columns were equilibrated with 4-(2-hydroxyethyl)-1-piperazineethanesulfonic acid (HEPES) buffer (10 mM HEPES, 50 mM NaCl, 1.6 mM KCl, 2 mM MgCl_2_, 3.5 mM CaCl_2_, pH to 7.4 with NaOH), removing the azide from the column. Two hundred microliters of Aβ in DMSO were then added to the equilibrated columns together with 40 μL of HEPES buffer as a stack and centrifuged (×1,000 *g*, 2 min, 4 °C). The eluted Aβ solution was collected in a LoBind Eppendorf tube (1.5 mL) on ice and the protein concentration was immediately measured via a NanoDrop spectrophotometer (280 nm, molar extinction coefficient of 1,490 cm^-1^M^-1^). Aβ was then immediately diluted to 50 μM in HEPES buffer and was left at room temperature for 2 h before adding it to the primary culture media at the desired concentration (1 μM). Filtered vehicle buffer was used as a control.

### Dissociated neuronal culture preparation

Rat primary hippocampal cultures were prepared from P_0_-P_1_ pups. Ethanol sterilized glass coverslips (12 mm, Fisher) were placed in 12 wells of a 24-well plate, and the remaining wells filled with sterile water. Poly-D-lysine (PDL, 20 μg/mL) was added to the wells and coverslips were incubated overnight at 37 °C, 5% CO_2_. The following day, PDL was removed, and coverslips dried completely before adding a laminin coating (20 μg/mL). Laminin was then aspirated before plating cultures. The hippocampi were dissected from both hemispheres, carefully removing any meninges. The dissecting solution was then removed, and the samples were washed 3 times with 2 mL of media (BME, 20 mM glucose, 10 mM HEPES, 1 mM sodium pyruvate, 2% FBS, 1% penicillin–streptomycin, 1% GlutaMAX, 2% B27) prewarmed at 37 °C. After the last wash, the tissue was gently triturated with a 1-mL pipette to yield a homogenous suspension. Cells were plated at a density of ~35,000 per well and were maintained at 37 °C, 5% CO_2_ in an incubator until they were used at DIV 14–18. To prevent excessive astrocytic growth, cultures were treated at DIV 4–6 with cytosine arabinoside (3.25 μM). For SV imaging experiments, sypHy was expressed by transfection using a calcium phosphate protocol (Promega Corp, Madison, WI, United States) at 7–10 days in vitro; iGluSnFR (pAAV-pCAG-iGluSnFr, Penn Vector Core) was expressed via transduction after 7 days in vitro.

### Fluorescence imaging

Imaging experiments were performed using an upright microscope BX61WI with a 60 × 1.0 NA dipping objective and Hg/Xe light source fitted with a cooled electron multiplying charge-coupled device camera (Andor Ixon, binning: 4 × 4, readout speed: 13,000 MHz, cooling temperature: −77 °C) or an Olympus XM10 CCD camera. Emission and excitation filters were 480/20 and 520/35, respectively. For each experiment, a coverslip was transferred from culture media to prewarmed extracellular bath solution (EBS, in mM: 137 NaCl, 5 KCl, 2.5 CaCl_2_, 1 MgCl_2_, 10 D-glucose, 5 HEPES, prewarmed to 37 °C) in a custom-built field stimulation chamber. CNQX (20 μM) and AP5 (50 μM) were added to the EBS to reduce recurrent network excitation and experiments were carried out at 23–25 °C. Stimulation was driven by a Grass SD9 controlled by custom-written Micromanager scripts or with the Olympus Cell-M experiment manager. Acquired image stacks were analyzed using FIJI and MATLAB or with Igor Pro (ver. 7.0, WaveMetrics) using the SARFIA plugin (Dorostkar et al. 2010).

### Acute slice preparation and synaptic labeling

Acute transverse hippocampal slices (300 μm) were prepared from APP_Sw,Ind_ or WT animals (3 months old) using a vibrating blade microtome and were then maintained in an artificial cerebrospinal fluid (ACSF) containing (in mM): 125 NaCl, 2.5 KCl, 25 glucose, 1.25 NaH_2_PO_4_, 26 NaHCO_3_, 1 MgCl_2_, 2 CaCl_2_, 20 μM CNQX, 50 μM AP-5 (bubbled with 95% O_2_ and 5% CO_2_, pH 7.3) (Staras et al. 2010; Rey et al. 2015, 2020). Experiments were performed at 28–29 °C and were controlled by a thermostatic inline heater perfusion system. To label synaptic terminals, a FM1-43FX-filled recording electrode (Molecular Probes, 20 μM in ACSF) (3–5 MΩ) was placed in hippocampal region CA1, and a bipolar tungsten stimulating electrode was positioned on the upstream Schaffer collaterals (SC). FM-dye was pressure applied for 3 min using a picospritzer to allow the dye to accumulate around the target region after which the SC were stimulated with a saturating stimulus protocol (20 Hz for 30 s, total: 600 APs) to label the recycled pool (Marra et al. 2012, 2014; Rey et al. 2020). Puffing of FM-dye continued for 2 min after the end of stimulation to ensure completion of endocytosis. Slices were then washed continuously in fresh ACSF for 20 min before fixing.

### Dye photoconversion and electron microscopy

Samples were fixed for 2 min using rapid microwave fixation (6% glutaraldehyde, 2% formaldehyde in PBS) (Marra et al. 2014; Rey et al. 2020). Samples were then transferred into 100 mM glycine (1 h), rinsed in 100 mM NH_4_Cl (1 min) and were then washed in PBS. For photoconversion, slices were placed on a dedicated photoconversion setup in an oxygen-bubbled diaminobenzidine solution (DAB, 1 mg/mL) and were visualized with a 40 × 0.8 N.A. dipping objective. The region of interest was identified from the position of the dye-containing pipette and was then illuminated with intense blue light from an Hg source (<500 nm) for 40 min. At the end of this period, slices were washed in PBS and then with ice-cold 0.15 M cacodylate buffer containing 2 mM CaCl_2_. For preparing samples for electron microscopy, we followed previously described methodology (Deerinck et al. 2010; Rey et al. 2015, 2020). Briefly, samples were placed on ice in a solution containing 3% potassium ferrocyanide in 0.3 M cacodylate buffer containing 4 mM CaCl_2_ that was mixed with an equal volume of 4% osmium tetroxide (1 h) and were then immersed sequentially in filtered warm 1% thiocarbohydrazide solution (20 min, room temperature), 2% osmium tetroxide (Sigma) (30 min, room temperature), and 1% uranyl acetate overnight at 4 °C. Next, samples were placed in lead aspartate solution in a 60 °C oven for 30 min and then successively dehydrated through graded ice-cold alcohols, and finally, into anhydrous acetone. Samples were flat-embedded in Durcupan resin (Sigma) between 2 ACLAR sheets and were trimmed to the central area in the photoconverted region. For sectioning, polymerized tissue blocks were mounted on the sample holder of a Leica EM UC7 ultramicrotome. The tissue was located within the block and an area of roughly trapezoidal shape was cut using a razor blade (Astra superior platinum double edge razor blade). To remove the superficial layer of resin present on the tissue, a glass knife was initially used. Once the tissue was exposed, ultrathin sections of ~60 nm were cut using a diamond knife (Ultra Diamond Knife—Wet 45^o^ 2.5 mm). Sections were collected on hexagonal 300-mesh Nickel grids (3.05 mm, Agar Scientific) and were imaged using a JEOL JEM1400-Plus Transmission Electron Microscope at 120 kV with a Gatan OneView 4 K CMOS digital camera.

### Electron microscope analysis

SV pools in the electron microscope (EM) images were quantified as described previously (Marra et al. 2012; Rey et al. 2015, 2020). Target synapses with PC+ vesicles were selected randomly, and each vesicle classified nonblind either as photoconverted (corresponding to recycled) or nonphotoconverted (resting) based on their vesicle lumenal intensity (Darcy et al. 2006a, 2006b). Analysis was limited to single section counts of <100 vesicles to ensure uniformity for comparison between groups. Spatial frequency density plots were generated using custom-written MATLAB routines by measuring vesicle coordinate positions and AZ structures. Middle-section electron micrographs were oriented so that the AZ was at the bottom and then the AZ geometry, as well as coordinates of each vesicle (either PC+ or PC−), were plotted. Synapse sections with multiple AZs visible were not included in the analysis. To represent vesicle positions, coordinates from all synapses were plotted on a grid relative to the center of the AZ in each case and were converted to a color-coded spatial density distribution. Vesicle positions were calculated to assume lateral symmetry around the midline (asymmetrical features are not informative because synapses are collected at all orientations in the slice). For display purposes, we doubled the intensity of pixels for recycled versus resting pools so that comparisons of spatial distribution could be readily made. Euclidean distances from each vesicle to its nearest point on the AZ were calculated to generate cumulative distribution plots. We defined the population of vesicles associated with the AZ as those that lay within 20 nm of the release site membrane and with a clear line of sight. In effect, this population corresponds to the first line of vesicles with access to the AZ. We did not attempt to define a morphologically docked pool since our images did not allow for unequivocal assessment of tethering structures that might link the AZ with vesicles. For cluster analysis, we measured the fraction of PC+ vesicles in concentric circular regions of interest of increasing size (20-nm radial distance steps) from the center of each PC+ vesicle, and values for these circular regions were expressed as the fraction of PC+ vesicles in the whole synapse. In other words, this clustering index tends to 1 as the size of the circle fully encompasses the whole population of vesicles in the terminal. Note, since this quantification only considers the vesicle “fraction” within this defined region, it is not influenced by the circle exceeding the boundaries of the terminal as it enlarges. To determine whether local clustering was significantly higher or lower than the baseline representation of PC+ vesicles in the whole terminal, we used one sample *t*-tests to compare each cluster index value at each distance against 1. We also examined possible relationships between the degree of clustering and the position in the synapse by dividing the population of vesicles into compartments (rear/side: 390–900 nm, middle: 250–390 nm, front: 100–250 nm, AZ: 0–100 nm) and then running a cluster analysis separately on these regions. To reveal differences in recycled vesicle pool spatial distributions in APP_Sw,Ind_ versus WT, we generated normalized versions of each plot (where total signal summed to 1) and calculated pixel-by-pixel differences between them.

### Statistics

Statistical comparisons used MATLAB or GraphPad Prism. Datasets were summarized as mean ± standard error of mean (SEM) or median and interquartile (IQR) range, as appropriate. Two-sample comparisons used 2-tailed unpaired or paired *t*-tests. One-sample *t*-tests were used to compare values against 1. Mann–Whitney U tests were used for comparing nonnormally distributed datasets. Correlation analysis used Spearman’s rank. Statistical significance was defined as *P* < 0.05.

## Results

### Elevated Aβ42 is associated with stable expansion of the TRP fraction in vitro

**Fig. 1:**
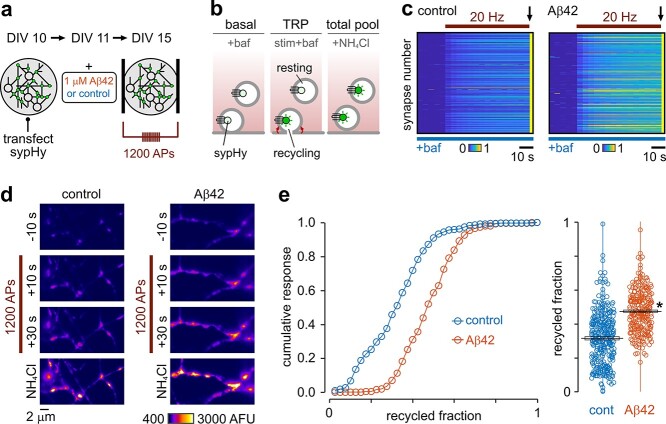
Expansion of the functional vesicle pool fraction with Aβ42 incubation. a) Schematic of approach. Experiments used dissociated hippocampal cultures expressing sypHy, a fluorescent probe that reports vesicle turnover. b) Alkaline trapping method. Synapses were field-stimulated (1,200 APs) in the presence of bafilomycin, a vATPase blocker, that reveals the full recycled vesicle pool. Washing synapses in NH_4_Cl was subsequently used to unmask the total vesicle pool, allowing recycling and resting fractions to be quantified. c) Plots show fluorescence responses for each synapse (horizontal lines) normalized to the NH_4_Cl response (arrow) for control and 1 μM Aβ42-treated cultures. d) Representative images showing fluorescence responses to stimulation and NH_4_Cl with control and Aβ42. e) Cumulative frequency distribution plot showing the recycled pool fraction comparison for control (0.32 ± 0.04, *n* = 343 from 7 preparations) and Aβ42-treatment (0.49 ± 0.02, *n* = 258 synapses from 6 preparations). Line and box show mean ± SEM. Asterisk indicates *P* = 0.0048, unpaired *t*-test.

We first considered whether elevated oligomeric Aβ42 impacts on the size of the vesicle pool available to undergo transmission. Neuronal cultures were treated with 1 μM Aβ42 oligomers (4–5-day incubation) (Fig. 1a) and functional vesicle pools were recruited by a saturating (1,200 AP 20 Hz) electrical field stimulus. We used the genetically encoded optical reporter, sypHy, a fusion construct of synaptophysin and pHluorin that reports the deacidification in the vesicle lumen occurring as it becomes exposed to the extracellular solution during exocytosis. Stimulation was carried out in the presence of 1 μM bafilomycin, a vATPase inhibitor that prevents vesicle reacidification and thus allows the full complement of functional vesicles (the TRP) to be unmasked in time-lapse images (Sankaranarayanan and Ryan 2001) (Fig. 1b). The second step in the protocol used 50 mM NH_4_Cl to alkalinize all vesicles, providing a measure of the total vesicle pool. In this way, an estimate of the functional pool size could be expressed as a fraction of the total number of vesicles at each terminal (Kim and Ryan 2010; Ratnayaka et al. 2012) (Fig. 1b). We found that this fractional size was highly variable across a synaptic population, which is consistent with previous work (Harata, Pyle, et al. 2001; Kim and Ryan 2010; Welzel et al. 2011; Ratnayaka et al. 2012). Nonetheless, Aβ42-treated synapses still exhibited a significant mean increase (~53%) in the recycling pool fraction versus controls (Fig. 1c–e). There was no significant change in total pool size, although there was a trend toward smaller values (control: 424 arbitrary fluorescence units [AFU], *n* = 343 from 7 preparations vs. Aβ: 303, *n* = 258 synapses from 6 preparations; unpaired *t*-test, not significant) such that, despite the increased recycling fraction, the absolute mean sizes of the recycling pool in Aβ42-treated versus control synapses were not different (control: 125 AFU vs. Aβ: 141, unpaired *t*-test, not significant). Our findings support the idea that while synapses exposed to Aβ42 have total vesicle pools of similar magnitude to those from untreated terminals, they dedicate a significantly larger fraction of these to support recycling.

### Ultrastructural assessment of vesicle pool size in a mouse model of AD

Next, we examined functional pool segregation in a transgenic AD model, tetO-APP_Sw,Ind_, reared without doxycycline treatment to produce developmental onset (Jankowsky et al. 2005; Sri et al. 2019). These animals show 20-fold and 7-fold increases in Aβ42 and Aβ40 production, respectively, compared to APP expression-matched mature-onset animals at 12 weeks of age (Sri et al. 2019).

**Fig. 2:**
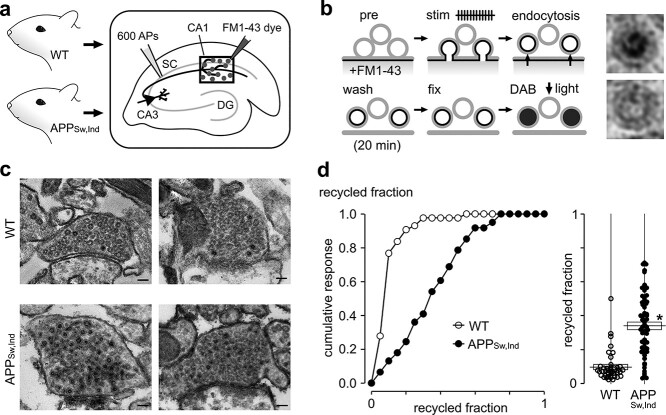
Expansion of the functional vesicle pool in APP_Sw,Ind_ mice. a, b) Schematics illustrate experimental approach. a) CA3-CA1 synapses in acute hippocampal slices taken from WT or APP_Sw,Ind_ mice were FM1-43 dye-loaded using electrical stimulation of SC. Dentate gyrus (DG). b) Synapses were stimulated in the presence of FM1-43 dye, washed, fixed, and photoconverted with photoillumination and DAB. Labeled and recycled vesicles (top, right) have a distinctive electron-dense lumen in electron micrographs versus unlabeled vesicles (bottom, right). c) Typical electron micrographs for activated WT and APP_Sw,Ind_ synapses in target region characterized by dark lumen and clear lumen vesicles. Scale bar: 100 nm. d) (Left) cumulative frequency distribution plot showing the recycled pool fraction comparison for WT (*n* = 43 synapses) and APP_Sw,Ind_ (*n* = 61 synapses). (Right) scatter plot for same datasets. Line and box show mean ± SEM (WT: 0.10 ± 0.01, APP_Sw,Ind_: 0.34 ± 0.02). Asterisk indicates *P* < 0.01, unpaired *t*-test.

To provide a readout of functional vesicle pool turnover and nanoscale characterization of pool organization that could offer detailed insights into the nature of synaptic deficits, we used a powerful function-ultrastructural assay developed previously in our lab (Marra et al. 2012, 2014; Rey et al. 2015, 2020). Specifically, we prepared acute hippocampal brain slices derived from APP_Sw,Ind_ and WT animals and labeled CA3 → CA1 synapses with the activity-dependent fluorescent dye FM1-43 (Betz and Bewick 1992; Ryan et al. 1993; Gaffield and Betz 2006; Cousin et al. 2018) using electrical stimulation of the upstream SC to recruit the recycling pool (TRP, Fig. 2a) (Zakharenko et al. 2001; Staras et al. 2010; Ratnayaka et al. 2011; Marra et al. 2012, 2014; Rey et al. 2015, 2020). After a 20-min period to allow recycled vesicles to endocytose and recluster, the tissue was fixed and the FM dye was used to drive the photoconversion of diaminobenzidine to produce an electron-dense precipitate that could be visualized at ultrastructural level (Henkel et al. 1996; Teng and Wilkinson 2000; Harata, Ryan, et al. 2001; Schikorski and Stevens 2001; de Lange et al. 2003; Rizzoli and Betz 2004; Darcy et al. 2006b; Denker et al. 2009, 2011) (Fig. 2b). This permits recycled vesicles to be discriminated from resting vesicles based on the dark luminal appearance of the former but not the latter, allowing us to quantify the size of the recycling pool as the proportion of total vesicles in each photoconverted terminal (Fig. 2b and c). Our results revealed that a significantly larger functional pool fraction was present in APP_Sw,Ind_ synapses versus WT with a broader spread of values (Fig. 2c and d). Consistent with our in vitro findings, this ex vivo ultrastructural approach supports the idea that the sustained presence of elevated Aβ42 is associated with a persistent expansion of the functional vesicle pool fraction.

**Fig. 3:**
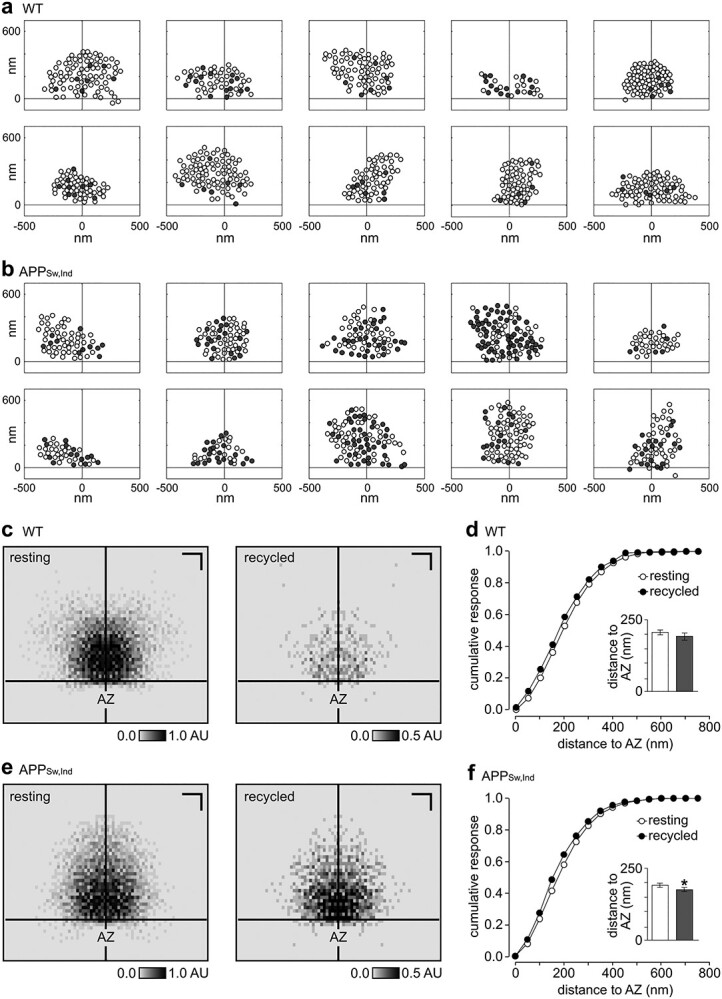
Organization of functional vesicle pools in APP_Sw,Ind_ synapses. a, b) Representative single-section cartoons of vesicle distribution for WT a) and APP_Sw,Ind_ b) synapses. Cross hairs indicate the AZ center. c) Normalized spatial frequency density plots showing the positioning of resting and recycled vesicles in WT synapses with respect to the AZ (*n* = 43 synapses). Scale bars, 200 nm. d) Cumulative frequency distribution plot for resting and recycled vesicles for distance to nearest point on AZ for WT synapses (*n* = 43). Inset shows mean ± SEM distances for each vesicle class (resting: 206 ± 8, recycled: 191 ± 13). e, f) as given in c, d) for APP_Sw,Ind_ synapses (*n* = 61 synapses). f) (Inset) shows mean ± SEM distances for each vesicle class (resting: 191 ± 8, recycled: 176 ± 7). Asterisk indicates *P* < 0.01, unpaired *t*-test.

### Nanoscale correlates of functional effects of Aβ42

The spatial organization of functional vesicles in presynaptic terminals is an established parameter that contributes to the efficacy of neurotransmission (Marra et al. 2012; Park et al. 2012; Rey et al. 2020). Next, we used the nanoscale detail offered by our functional-ultrastructural method to investigate whether the APP_Sw,Ind_ genotype influenced the arrangement of vesicles within the presynaptic terminal architecture. Based on representative middle sections for each synapse (Fig. 3a and b), we first mapped the coordinates of all recycled and resting vesicles with respect to the AZ and summarized them in mean distribution density plots for both pools in each synapse. These plots were then overlaid, smoothed, and color-coded based on vesicle density. In WT synapses, we found that resting vesicles formed a homogeneous density cloud while recycling vesicles clustered at sites closer to the AZ (Fig. 3c). To provide direct quantification of our findings, we measured raw Euclidean distances between each vesicle and the nearest point on the AZ and plotted cumulative frequency distribution plots and distance distribution plots for each pool class (Fig. 3d). Consistent with the density maps, recycling vesicles in WT synapses favored positions closer to the AZ, observed as a left shift in the cumulative plot versus the resting pool. This bias in recycling versus resting vesicles aligns with previous observations (Marra et al. 2012), showing that recycled vesicles return to preferential locations in the pool architecture with respect to the AZ. Next, we carried out the same analysis in APP_Sw,Ind_ samples. Strikingly, although the fraction of recycled vesicles was much higher in APP_Sw,Ind_ synapses, the relative distribution was similar to those in WT synapses; again, recycled vesicles took up positions closer to the AZ than resting vesicles (Fig. 3e and f). Thus, in broad terms, the population of recycled vesicles occupies approximately the same relative space within the cross section of the terminal.

**Fig. 4:**
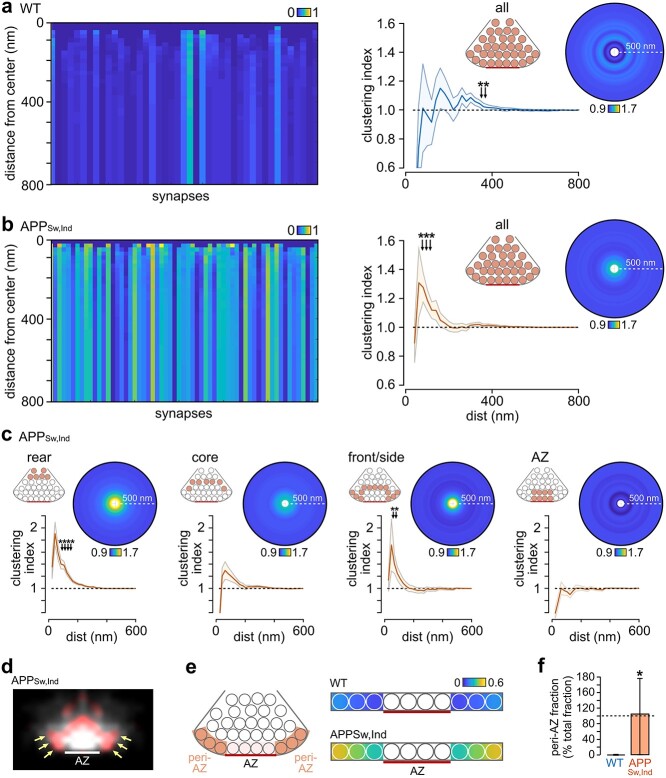
Local clustering of recycled vesicles in APP_Sw,Ind_ mice and accumulation at the peri-AZ. a) (Left) color-coded plot of mean absolute recycling fractions with distance away from the center of PC+ vesicles for WT synapses; each synapse is represented by a vertical bar. Color scale indicates recycling fraction. (Middle) mean ± SEM plot of clustering index with increasing distance from vesicle center (*n* = 43 synapses). Asterisks indicate significant clustering (1-sample *t*-tests vs. 1, *P* < 0.05). (Right) mean circular frequency density plots showing relative PC+ clustering normalized to final recycling fraction for the whole cluster. Color scale indicates clustering index. b) As in a) for APP_Sw,Ind_ synapses (*n* = 61 synapses). c) Mean ± SEM plot and circular heatplots of clustering index with increasing distance from vesicle center for compartments of APP_Sw,Ind_ terminals; cartoons indicate the different regions in the terminal. d) Heatplot showing clustering sites of recycled vesicles with Aβ42 treatment. Red signal is the recycled vesicle distribution in Aβ42-treated synapses after the control profile has been subtracted, indicating that there is accumulation of vesicles at the peri-AZ region. White signal is the recycled vesicle distribution without subtraction. e) (Left) schematic of region of synapse used for compartment analysis. We identified the 3-vesicle area adjacent to the AZ on each side and scored the vesicle occupation at these sites. (Right) heatplot summary indicates the relative occupation of these regions by recycled vesicles in WT and APP_Sw,Ind_ synapses. f) Quantification of median ± IQR range for recycled vesicle occupation in the peri-AZ, normalized for the difference in recycled fraction for WT and APP_Sw,Ind_ terminals (*P < 0.001, Mann–Whitney U test, WT: 0% (0–0), *n* = 35) 106% (0–179), *n* = 56).

To provide a finer-grade description of vesicle organization, we next carried out a cluster analysis, which quantifies how recycled vesicles are positioned relative to each other and provides insights into potential differences in postendocytic trafficking pathways between WT and APP_Sw,Ind_. Recycled vesicle fractions were measured in concentric circular regions of interest of increasing size (20-nm radial distance steps) surrounding each individual recycled vesicle. In Fig. 4a and b (left panels), each vertical bar represents the mean absolute recycling fraction for each synapse as the distance from the center of each recycled vesicle increases, with the variation in intensity between WT and APP_Sw,Ind_ reflecting the absolute differences in their recycled fractions. Next, we normalized this clustering index to the total recycled fraction at each synapse, allowing changes in clustering to be considered independently of the observed difference in recycling fraction (Fig. 4a and b, right panels). In WT animals, these plots revealed no significant short-distance clustering (1-sample *t*-tests vs. 1). However, local clustering was a significant feature of APP_Sw,Ind_ synapses, seen as a distinct local peak (80–120 nm) in the cluster plot; in other words, recycled vesicles in APP_Sw,Ind_ synapses tend to accumulate at close distances in the terminal.

Next, we examined the source of this accumulation of vesicles by running cluster analysis selectively on different compartments in the synapse (rear: 390–900 nm, core: 260–390 nm, front/peripheral: 130–260 nm, AZ: 0–130 nm, Fig. 4c). This approach revealed two specific regions where clustering was prominent, one at the rear and a second in the area behind and lateral to the AZ, including the peri-AZ (Fig. 4c), a site of functional relevance as the proposed location for vesicle retrieval following fusion (Roos and Kelly 1999; Slepnev and De Camilli 2000). To provide further support for this finding, we overlaid normalized recycled pool heatplots of WT and APP_Sw,Ind_ (see Fig. 3a and c) so that we could compare point-by-point differences in their distributions. Consistent with our local clustering results, this indicated that peri-AZs were hotspots for recycled vesicle accumulation in APP_Sw,Ind_ (Fig. 4d). For quantification of this, we returned to the EM images and carried out a compartment analysis, identifying the lateral zones in each synapse and scoring the vesicle occupation at these sites. The number of recycled vesicles was significantly elevated in peri-AZs in APP_Sw,Ind_ versus WT, with the highest accumulation at the most distal region from the AZ (Fig. 4e). Even when we corrected for the higher recycled fraction in the synapse as a whole, APP_Sw,Ind_ terminals showed a significantly elevated recycled vesicle occupancy in this region (Fig. 4f). Our findings indicate that recycled vesicles excessively accumulate at putative retrieval sites in the APP_Sw,Ind_-elevated Aβ model, consistent with the idea that retrieval and trafficking mechanisms may be defective.

### Functional consequences of Aβ42 for ongoing signaling and rescue

**Fig. 5:**
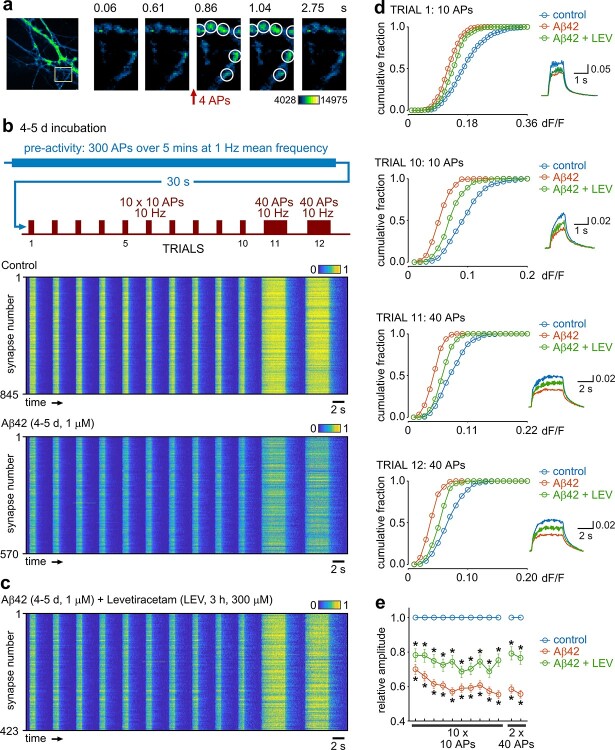
Glutamate transmission defects after low-frequency activity with 4–5-day Aβ42-treatment and partial rescue. a) Representative images showing iGluSNFR signal and response to stimulation. b) Color-coded response heatplots (normalized dF/F) to the stimulation paradigm outlined in schematic (top) for control and Aβ42-treated dissociated cultured neurons. The recorded stimulation protocol was preceded by a 5-min low-frequency activity paradigm consisting of repeated bouts of 5-s stimulation (2 Hz) and 5-s rest. Horizontal lines represent individual synaptic terminals with values normalized to the mean trial-by-trial control responses. c) As in b) but with LEV (300 μM, 3 h). d) Cumulative distribution plots of amplitudes for trials 1, 10, 11, and 12 (see protocol). Inset shows mean response for control (*n* = 845 synapses from 4 preparations), Aβ42 (*n* = 570 from 4 preparations), and Aβ42 + LEV (*n* = 423 from 4 preparations). e) Summary of mean ± SEM amplitude for Aβ42 or Aβ42 + LEV normalized to control amplitude for each trial. Asterisks indicate significance (*P* < 0.05, unpaired *t*-tests based on comparisons of raw data).

If excess Aβ42 impairs vesicle recycling pathways, this might have profound consequences for ongoing functional transmission. To test this idea, we expressed iGluSnFR, a genetically encoded optical probe that reports the extracellular glutamate release associated with synaptic signaling at excitatory terminals as a transient rise in fluorescence (Fig. 5a) (Marvin et al. 2013) in vehicle control or 1 μM Aβ42-treated (4–5-day incubation) cultured hippocampal neurons. Prior to testing transmission effects, we imposed a stimulation pattern that mimicked the typical firing frequency of neuronal spiking patterns reported in vivo (Mizuseki et al. 2012; Kowalski et al. 2016), allowing us to explore the activity-history dependency of Aβ42-associated signaling changes with physiologically relevant activity. iGluSnFR responses were then assayed with 10 × 10 APs (10 Hz) and 2 × 40 APs (10 Hz) stimulation (Fig. 5b). Compared to control cultures, Aβ42-treated neurons showed a significant reduction in the initial response amplitude that followed the baseline stimulus train (trial 1: ~70% of control) and a further pronounced reduction in response amplitude with subsequent rounds of stimulation (trial 10: ~55% of control) (Fig. 5b and d). Our findings are consistent with the idea that elevation of Aβ42 profoundly influences presynaptic glutamate signaling in a use-dependent manner.

Taken together, our results suggest that elevated Aβ42 drives initially excessive stimulus-driven recruitment of vesicles but that efficient retrieval and reuse become impaired with sustained activity perhaps reflecting an overwhelming of recycling pathways. We hypothesized, therefore, that dampening hyperactivity in synapses might help to restore ongoing signaling function. To test this, we examined the impact of LEV, an antiepileptic seizure agent that suppresses SV release in an activity-dependent manner (Meehan et al. 2011), on Aβ42-treated neurons. LEV has proposed therapeutic value in AD models (Sanchez et al. 2012; Tse 2012; Bakker et al. 2015), but the basis for these effects remains enigmatic. Strikingly, using the same iGluSnFR approach, LEV partially restored glutamate signaling toward control levels in Aβ42-treated synapses (Fig. 5c–e), consistent with the idea that targeting excessive vesicle turnover may have therapeutic potential.

## Discussion

Presynaptic dysfunction is thought to underlie cognitive deficits associated with a variety of brain disorders, yet SV pools remain largely unexplored targets (Li and Kavalali 2017). Here, we report their modulation in two models of AD, adding to an accumulating body of work implicating their likely involvement in forms of AD pathology (Kelly and Ferreira 2007; Puzzo et al. 2008; Abramov et al. 2009; Chakroborty et al. 2012; Russell et al. 2012; Park et al. 2013, 2017; Lazarevic et al. 2017; Marsh et al. 2017; Marsh and Alifragis 2018; Anni et al. 2021). Specifically, we focused on elucidating how organizational principles of functional vesicle pools—their size and spatial positioning in terminals—were influenced by elevated Aβ. Such pool characteristics are now well established as correlates of synaptic efficacy (Murthy et al. 1997; Branco et al. 2008, 2010; Branco and Staras 2009; Marra et al. 2012; Park et al. 2012; Rey et al. 2020) and thus are highly relevant to consider in generating a complete picture of pathology-linked synaptic dysfunction.

The present study used two main approaches: optical synaptic readouts in an Aβ42-elevated hippocampal model in vitro, and a function-ultrastructure assay in a chronic mouse model of elevated Aβ (APP_Sw,Ind_) ex vivo. The oligomeric Aβ42 treatment length (4–5 days) that we used in our in vitro preparation is longer than in some other studies but was chosen to reflect a persistent state of increased Aβ42 load and thus gives us the opportunity to study steady-state responses, arguably paralleling more closely our APP_Sw,Ind_ model. We found that the recycled fractional pool size increased in Aβ-exposed synapses, while the total vesicle pool size was similar or even marginally reduced. In other words, while Aβ-treated terminals have slightly smaller total SV pools, they dedicate a larger fraction of them to support recycling. A similar finding emerged from our nanoscale characterization of synapses in the APP_Sw,Ind_ model. Here, we also found that retrieved vesicles excessively accumulated at regions lateral to the AZ, a physical spatial correlate of impaired recycling. Because these regions are the likely locations where vesicles are recovered during endocytosis (Roos and Kelly 1999; Slepnev and De Camilli 2000), it is consistent with a potential stalling or overwhelming of the trafficking pathways that would normally return vesicles back into the vesicle cluster core prior to rerelease. The fact that we found a higher vesicle fraction recruited by a given stimulus in APP_Sw,Ind_ versus wild-type animals would thus place more vesicles in this endocytic pathway and would likely serve to exacerbate this overwhelming effect. We hypothesize, therefore, that the accumulation of recently retrieved vesicles with elevated Aβ might potentially be an obstacle to their efficient reuse in ongoing transmission. It is also conceivable that some of these retrieved structures could even be endolysosomal intermediates bound for a degradative fate rather than for a recycling one (Ivanova and Cousin 2022). The idea that there are deficits for ongoing signaling is supported functionally in experiments where we used the optical reporter, iGluSnFR, to directly monitor glutamate release. We show that impairment accumulates with repeated rounds of activity, presumably as stalled vesicle recycling pathways reduce the availability of transmitter-filled vesicles to undergo rerelease. Our results also hint at a possible disconnect between levels of vesicle fusion and transmitter release in Aβ42-treated neurons perhaps reflecting a defect in glutamate refilling mechanisms such that partially full or empty vesicles are undergoing recycling.

Our findings are consistent with a mechanism in which increased vesicle recruitment in a comparable or smaller total vesicle pool, alongside defective retrieval and reclustering, serve to limit sustained signaling when Aβ is significantly elevated. This suggests that strategies aimed at dampening excess activity-evoked vesicle fusion, or improving the efficiency of vesicle retrieval, might have therapeutic value. LEV, an antiepileptic agent that has a suppressing effect on network activity (Sanchez et al. 2012), has already been reported to have beneficial consequences for cognitive function in rodent and human AD models (Sanchez et al. 2012; Tse 2012; Bakker et al. 2015), although its precise mode of action remains to be clarified. In our study, LEV is effective in partially restoring glutamate transmission toward control levels. One possible explanation is that it functions by limiting hyperactivity in presynaptic terminals and therefore excessive turnover of vesicles, including, for example, those that might be defective for glutamate transmission, thus relieving an overload of vesicle retrieval pathways that would inhibit ongoing signaling. Collectively, our findings suggest that agents which help to support the efficient retrieval and reuse of recycling vesicles at small central terminals might have potential value as a therapeutic strategy to consider further in AD.

Direct comparisons between different AD studies are often problematic due to significant disparities in the choice of model, assay type, and specific Aβ concentrations and incubation periods they use. Nonetheless, the Aβ-linked susceptibility of functional vesicle pool segregation that we report here has broad parallels with other important studies demonstrating pool changes in elevated Aβ conditions (Kelly and Ferreira 2007; Park et al. 2013; Lazarevic et al. 2017). This could implicate CDK5 and calcineurin, known regulators of vesicle pool segregation (Kim and Ryan 2010; Marra et al. 2012), as potential substrates to mediate these changes, an idea that is already supported in the literature (Lazarevic et al. 2017; Anni et al. 2021). In our study, using long-term or chronically elevated Aβ, we did not see an immediate negative impact on vesicle recruitment. However, the deficits we report in postendocytic trafficking of vesicles are compatible with previous work suggesting that slowed vesicle retrieval and/or reacidification kinetics accompany subchronic Aβ treatment (Park et al. 2013). Our results, linking organizational changes in vesicle pools to synaptic pathology, also have parallels with other recent studies looking at different disorders. In a Huntington’s disease model, researchers used a “tour de force” real-time, 3-dimensional tracking readout in cortical synapses to show that altered vesicle movement dynamics accompanied presymptomatic disease, linked to Rab11 expression and actin filament disruption (Chen et al. 2021). Likewise, significant changes in the physical positioning of vesicles in terminals were reported in a neocortical epilepsy model, which was proposed to be a homeostatic response that might serve to reduce network synchronicity in the chronic phase (Vannini et al. 2020). Collectively, these findings suggest that vesicle pool dynamics and the nanoscale positioning of functional vesicle pools are emerging as important potential substrates in disease pathogenesis.

## Conclusion

Synaptic dysfunction is one of the earliest deficits observed in Alzheimer's disease (AD). Here we show that incubation with exogenously applied oligomeric Aβ influences vesicle recycling and is associated with impaired glutamate signalling. A transgenic model of AD that over-expresses Aβ provides evidence for specific changes in the size and spatial organization of vesicle pools at release sites. In cultured neurons, pharmacological intervention to limit aberrant vesicle trafficking partially rescues impairments in presynaptic signalling, highlighting this as a potentially promising therapeutic strategy.

## Authors’ contributions

L.B., M.F., S.R., A.R., K.F., C.S., and K.M. collected and analyzed the data. C.H. and M.V.-C. established and supported the APP_Sw,Ind_ mouse model work. K.S. analyzed some of the data and provided the figures. L.S. and K.S. directed the work and wrote the initial draft of the manuscript. All authors commented on the drafts of the manuscript.

## Data Availability

Datasets supporting this article are openly available from the University of Sussex Research Data Repository Figshare, and can be accessed at: https://doi.org/10.25377/sussex.19419539.
